# Wound Dressing Selection Is Critical to Enhance Platelet-Rich Fibrin Activities in Wound Care

**DOI:** 10.3390/ijms21020624

**Published:** 2020-01-17

**Authors:** Cristina Del Amo, Arantza Perez-Valle, Elena Perez-Zabala, Karmele Perez-del-Pecho, Ainara Larrazabal, Andima Basterretxea, Paola Bully, Isabel Andia

**Affiliations:** 1 Bioprinting Laboratory, Regenerative Therapies, BioCruces Bizkaia Health Research Institute, Cruces University Hospital, 48903 Barakaldo, Spain; cristina.delamomateos@osakidetza.eus (C.D.A.); arantza.perezvalle@osakidetza.eus (A.P.-V.); mariaelena.perezzabala@osakidetza.eus (E.P.-Z.); mariacarmen.perezpechodel@osakidetza.eus (K.P.-d.-P.); ainara.larrazabalarbaiza@osakidetza.eus (A.L.); Andima.basterretxeaozamiz@osakidetza.eus (A.B.); 2 Hospital-at-home Service, Cruces University Hospital, 48903 Barakaldo, Spain; 3 “Paola Bully”, Statistical and Methodological Consulting, 48190 Sopuerta, Bizkaia, Spain; paola.bully01@gmail.com

**Keywords:** regenerative medicine, platelet rich plasma, platelet rich fibrin, biomaterials, dressing, wound healing, cell activities

## Abstract

The use of platelet-rich fibrin (PRF) is investigated in ulcer management because it provides a healing milieu rich in growth factors and cytokines. Although crucial, the relevance of secondary dressings is under-researched and no data support the use of any particular dressing in preference to another. We assessed the properties of different dressing categories, including alginates, hydrocolloids, foams, hydrofibers, films, meshes and gauzes, in terms of affinity for PRF, releasate management (retention/extrusion) and the kinetics of cytokine release as well as the influence of each combination product, [PRF + dressing], on dermal cell behaviour, aiming to provide useful information for choosing the most adequate dressing for each particular patient. Active dressings including alginates, hydrofibers, foams and hydrocolloids blend with PRF, creating a diverse combination of products with different performances. Alginate and hydrofiber showed the highest affinity but moderate retention of releasate, without interfering with cell functions. Instead, the foam sequestered the releasate and hindered the release of growth factors, thereby compromising cell activities. Film and mesh presented very poor releasate retention and performed similarly to PRF by itself. Affinity index and releasate management explained 79% of platelet-derived growth factor (PDGF-BB) concentration variability, *p* < 0.001. Cell proliferation depended on the ability of the combination product to retain/release supernatant, PDGF-BB concentration and cell adhesion R^2^ = 0.91, *p* = 0.014.

## 1. Introduction

Research in the last three decades has resulted in improvement of wound care through the use of regenerative medicine technologies (i.e., stromal vascular fraction of adipose tissue (SVF), platelet-rich plasmas (PRPs), bone marrow concentrate (BMC)), which can boost repair mechanisms in stagnant injuries [1]. Such research efforts are primarily focused on reducing the economic and social burden generated by difficult-to-heal wounds that can turn into chronicity, such as diabetic foot ulcers, vascular ulcers, pressure ulcers, and surgical and necrotic wounds [2,3].

In physiological healing, platelets are involved in several biological processes, which take place in ordered spatiotemporal sequences; the initial hemostatic phase involves, in parallel, thrombin formation and platelet activation and aggregation. Upon degranulation, platelets release a pool of chemokines, cytokines and growth factors; specifically, platelets and plasma molecules can mediate inflammation, angiogenesis and the formation of extracellular matrix [4]. The research interest in blood-derived products for tissue repair is grounded in augmenting these mechanisms.

The use of platelets in chronic wounds dates back to the early 1990s [5]; preliminary clinical studies analyzed the efficacy of platelet-derived wound healing factors (i.e., PDWHF), i.e., the platelet secretome. The strategy was to provide a molecular healing milieu, including chemotactic cytokines to facilitate cell infiltration into the wound area (e.g., CXCL7 (neutrophil activating peptide), RANTES (regulated upon activation, normally T-expressed and presumably secreted), PF4 (platelet factor 4), SDF-1α (stromal cell derived factor 1) and growth factors, such as PDGF (platelet derived growth factor), EGF (epidermal growth factor), TGF-b1 (transforming growth factor), VEGF (vascular endothelial growth factor), and HGF (hepatocyte growth factor) to drive cell proliferation and angiogenesis [6]. This panel of active molecules released by platelets upon activation drives the healing activities of different cell phenotypes, including mesenchymal stem cells [7], innate immune cells and local cells [8,9]. In addition, the platelet secretome contains peptides and proteins (e.g., thrombocidins) with antibacterial activities [10]. Thus, current concepts in wound healing pathophysiology and PRP biology support the use of PRF as a multifunctional wound dressing hydrogel with the ability to release a large pool of healing molecules.

Currently, multiple devices and protocols to prepare PRP and other blood derivatives, such as platelet rich fibrin (PRF), have been commercialized and standardization efforts have created a framework for product description [11]. PRF can be described as a dynamic multifunctional hydrogel to be used as an active wound dressing. Essentially, upon calcium and/or thrombin addition, PRP forms a platelet-rich fibrin scaffold (PRF) that can be layered in the wound bed. PRF degradation is highly regulated by the serine protease system from plasma PAI-1, PAI-2 (plasminogen activator inhibitor type 1 and 2), TAF1 (TATA-box binding protein, associated factor), plasmin [12,13] and can be synchronized with the healing process.

PRF is adhesive, but when grafted in the wound bed, it changes its configuration and mechanical properties over time, i.e., retraction of the fibrin matrix and extrusion of the platelet secretome containing most of the signaling molecules. These mechanisms can be controlled by the combination of PRF with particular secondary dressings. However, it is unclear which secondary dressing enhances PRF activities.

According to a recent systematic review and meta-analysis, PRPs can be good candidates for managing chronic nonhealing ulcers [14,15], while uncertainty remains high in vascular ulcers. The results look promising, but there is a large variability in wound care protocols. Indeed, there are several wound dressing categories, including alginates, hydrocolloids, foams, hydrofibers, films, meshes, and gauzes and, although crucial, no data support the choice of any particular dressing in preference to another. In addition, collagen-based wound dressings (e.g., from bovine, porcine or avian origin) can help to manage chronic wounds by their participation in various healing stages.

To decide on the best secondary dressings for wound healing in PRF treatments, we examined the interactions of the above mentioned dressing categories with PRF grafts in terms of: (i) affinity for PRF (compactness of the combination product), (ii) ability in handling PRF releasate (retain or expel the cytokine rich supernatant), and (iii) control the release profile of active growth factors. Furthermore, we evaluated the performance of composite products, i.e., [PRF + dressing] through dermal fibroblasts functional assays. Our data aim to support and help the clinical practitioner in the decision of choosing the most adequate dressing category to be used with PRF.

## 2. Results

### 2.1. PRP and PRF-Releasate Characterization

The characterization of PRP and PRF releasate in cellular and molecular terms are shown in Table 1 and Table 2, respectively.

### 2.2. PR-Fibrin Stability

The PRF stability decreased over time, *p* < 0.001 (repeated measures ANOVA).

The mean PRF stability was 24.77% ± 4.21% (95% CI: 21.94–27.60) at 3 days; 22.01% ± 5.73% (95% CI: 18.16–25.87) at 4 days; 20.07% ± 5.29% at 5 days (*n* = 11) and 18.11% ± 5.76% at 7 days (*n* = 11). The PRF stability was not influenced by the blood donor.

### 2.3. Affinity for PRF Differed Depending on Wound Dressing Composition

The affinity index for PRF depended on the biomaterial that composed the dressing. Alginate and hydrofiber showed the highest affinity for PRF (14.00 ± 0.11 and 13.45 ± 0.22); foam and gauze had moderate PRF affinity (8.37 ± 0.92 and 8.42 ± 0.42, respectively). The affinity index of mesh was 4.57 ± 1.31 and the hydrocolloid had an affinity index of 2.33 ± 0.30; as expected, the film showed no affinity for PRF (0.200 ± 0.209).

**Figure 1:**
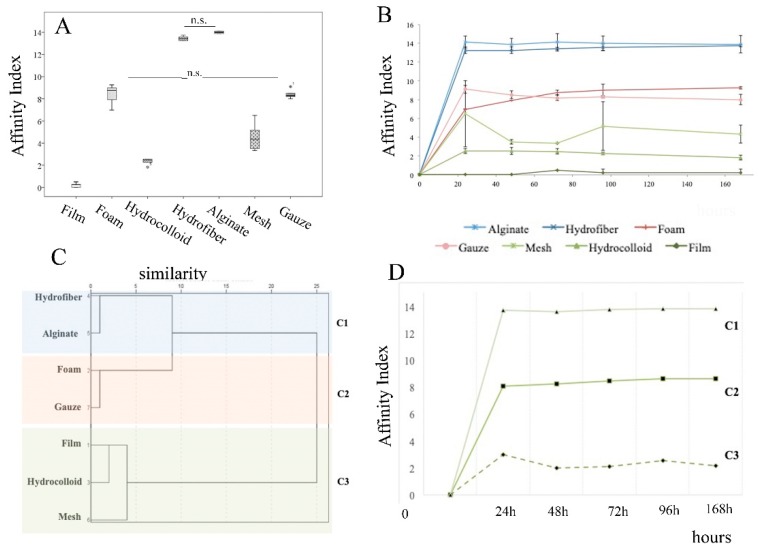
(**A**) The affinity index for PRF differed between dressings, *p* < 0.001 for all comparisons except “hydrofiber versus alginate”, and “foam versus gauze”, which were non-significant (n.s.) differences. Box plots depict the median, lower and upper quartile, symbols outside the box represent outliers; (**B**) Affinity index for PRF graft based on weight changes of the combination product, i.e., [PRF + dressing], over 7 days; (**C**) Dendogram showing the hierarchical relationship between dressings, the X axis is a measure of closeness of either individual dressings or clusters; cluster analyses reveal three groups of dressings, C1 (blue background) C2 (pink background), and C3 (blue background) (**D**) Cluster differences in the affinity index measured over 7 days.

Cluster analysis discriminated three dressing categories. Alginate (Melgisorb^®^ Plus) and hydrofiber (Aquacel^TM^ Extra^TM^) were grouped in the same category. The second cluster included foam and gauze, while film, mesh (Tegaderm^™^ Film and Acticoat^®^ Flex 3) and hydrocolloid (Varihesive^®^ Gel Control) were clustered together with scarce affinity for PRF (Figure 1).

### 2.4. PRF Releasate Management

**Figure 2:**
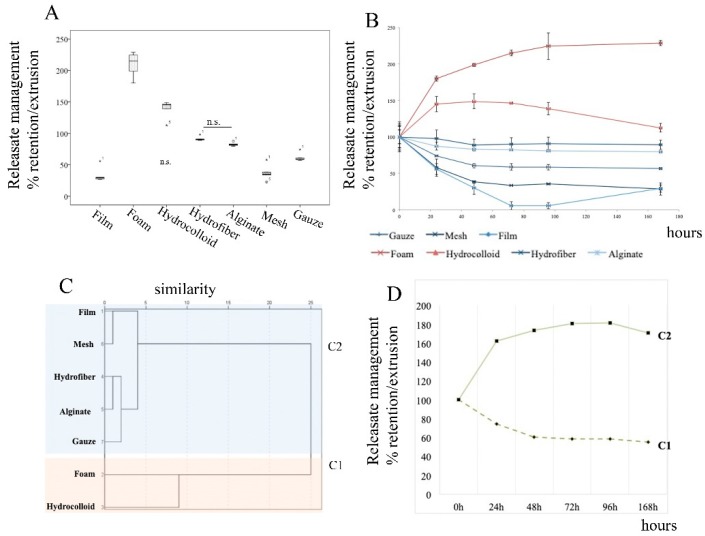
(**A**) Releasate management (uptake/release) differed between dressings, *p* < 0.001 for all comparisons except “hydrofiber versus alginate” and “film versus mesh”, which were non-significant (n.s.) differences. Box plots depict the median, lower and upper quartile, symbols outside the box represent outliers; (**B**) Percent retention/extrusion of the releasate over 7 days; (**C**) Dendogram showing the hierarchical relationship between dressings, the X axis is a measure of closeness of either individual dressings or clusters; cluster analyses reveal two groups of dressings, C1 (pink background) and C2 (blue background); (**D**) Cluster differences in releasate management measured over 7 days.

Management of the releasate (i.e., retention/extrusion) varied upon the dressing’s composition (Figure 2A,B). The foam and the hydrocolloid showed strong retention of the releasate, while the hydrofiber, alginate and gauze showed moderate retention. The film and the mesh showed no retention at all (similar behavior as PRF).

Cluster analysis discriminated two main groups (Figure 2C,D). The foam (Mepillex^®^ Border) and the hydrocolloid (Varihesive^®^ Gel Control) showed very robust releasate retention and clustered together; both retained a greater quantity of liquid than their initial weight. Within this group, the ability to retain the releasate was higher for the foam, (209.37% ± 20.07%, 95% CI 184–234), compared to the hydrocolloid (138% ± 14.89%, 95% CI 119–157) (*p* < 0.001). On the other hand, alginate or hydrofiber (Melgisorb^®^ plus and Aquacel™ extra ™, respectively) extruded the PRF releasate in a stable and similar mode over time, (91% ± 3% and 83% ± 3%, respectivezly). The gauze retained 58.71% ± 1.65% of the releasate (Figure 2).

Combination products made with film/mesh dressings (e.g., Tegaderm^™^ Film and Acticoat^®^ Flex 3) presented very poor releasate retention and performed similarly to PRF by itself.

### 2.5. PDGF-BB Release Kinetics

**Figure 3:**
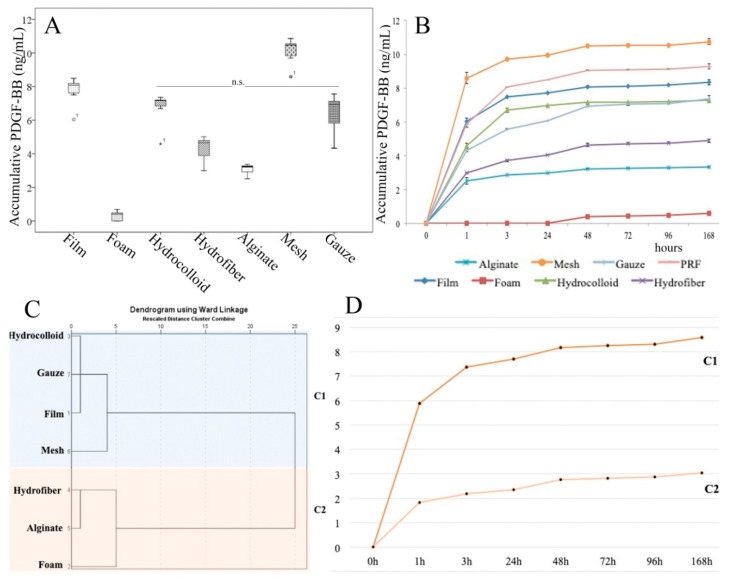
(**A**) Released PDGF-BB differed between dressings, *p* < 0.001 for all comparisons except “hydrofiber versus alginate”, non-significant differences (n.s.) Box plots depict the median, lower and upper quartile, symbols outside the box represent outliers; (**B**) Pattern of PDGF-BB release over 7 days; (**C**) Dendogram showing the hierarchical relationship between dressings, the X axis is a measure of closeness of either individual dressings or clusters; (**D**) Cluster differences in the PDGF-BB release measured over 7 days.

Figure 3 shows the pattern of PDGF-BB release for all combination products. Most of the release of PDGF-BB from the combination products occurred within the first 24 h. Of note, [PRF + Foam] did not deliver any PDGF-BB.

There was a significant inverse correlation between releasate management (% retention/extrusion-ejection) and PDGF-BB concentration (r = −0.764, *p* < 0.001) and a significant inverse correlation between affinity index and PDGF-BB (r = −0.606, *p* < 0.001). Instead, there was no significant correlation between releasate management and affinity index (r = 0.191 *p* = 0.273).

Linear regression analyses revealed that the combination products’ properties, i.e., affinity index and releasate management, explained 79.1% of the PDGF-BB concentration variability (R^2^ = 0.791, PDGF_conc_ (ng/mL) = 11.428 − 0.302 × affinity index-0.036 × releasate management).

### 2.6. Cell Experiments

**Figure 4:**
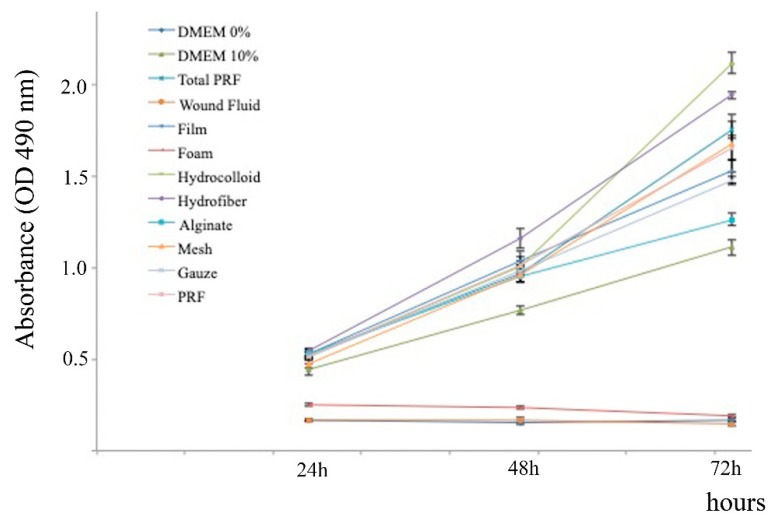
Comparative analysis of the effect of the different combination products’ releasates in the cell proliferation of dermal fibroblasts, measured at 24, 48 and 72 h. The XTT (2,3-bis(2-methoxy-4-nitro-5-sulfophenyl)-5-[(phenylamino) carbonyl]-2H-tetrazolium hydroxide) cell proliferation assay is a colorimetric assay, based on cell metabolic activity, for assessing cell growth and division. In particular, the number of cells present in a condition directly correlates with the absorbance obtained. Of note, the combination product [PRF + Foam] and the simulated wound fluid (WF) exhibit similar behavior, i.e., no proliferation, comparable to the behavior of fibroblast incubated in DMEM without serum.

Releasates from all the combination products, except the releasate from [PRF + Foam], induced dermal fibroblast proliferation (Figure 4). The combination product [PRF + Foam] hindered cell viability after 72 h of culture.

Regression analyses showed that cell proliferation depended on the ability of the combination product, i.e., [PRF + dressing categories], to retain/release the supernatant (“releasate management”), PDGF-BB concentration, and cell adhesion, R^2^ = 0.91 F = 23.19 *p* = 0.013. Instead, we could not find any relationship between cell migration and combination products (data not shown).

## 3. Discussion

The results of this study were expected to provide pragmatic information that could be translated to the clinic regarding the choice of the most appropriate secondary dressing to be combined with the PRF graft for wound healing. Despite the expansion of PRP therapies in ulcer care, the choice of secondary dressings is under-researched.

In fact, active dressings, including alginates, hydrofibers, foams, and hydrocolloids blend with PRF, creating diverse combination products with different performances. The macroscopic changes include thickening or shrinking and changes in stability over time. Using the current in vitro model, i.e., maintaining combination products in inserts for seven days in cell culture conditions, with artificial wound fluid at the bottom of the well, we evaluated the differences between dressing categories. First, we assessed the affinity index to obtain data on the compactness of different combination products, [PRF + dressing]. On the other hand, we examined the ability of the combination products to retain or expel the supernatant rich in cytokines and growth factors, using PDGF assessments as representative of alpha granule cargo exocytosis [16]. To prepare the PRF, we pooled PRPs from twelve subjects; we chose this model to obtain meaningful reproducible results, getting rid of inter-donor variability [17,18].

As a representative molecule in the context of wound healing, we assessed PDGF-BB concentration in each ejected supernatant. PDGF-BB is stored in platelets’ alpha granules, thus it is representative of platelet degranulation and involved in the pathophysiology of chronic ulcers [19,20,21]. Indeed, the initial use of platelets in wound healing was to some extent based on the presence of platelet-derived growth factor (PDGF-BB). Recombinant human PDGF-BB, rh-PDGF-BB (becaplermin, (Regranex), J&J, NJ), is considered a potent wound healing factor in chronic pressure ulcers [19] and diabetic foot ulcers [20] and was cost effective over standard care in the management of pressure injuries [21].

Since dressings do not contain cell-signaling molecules, their combination with PRF provides autologous PDGF-BB as well as a large pool of cytokines involved in healing mechanisms [22]. All of the combination products we created had an initial burst (within three hours) followed by a slow release of PDGF-BB over 7 days. According to our study results, alginates, hydrofibers, foams, and hydrocolloids have different growth factor uptake capacities. Hydrofiber and alginate, as well as hydrocolloids, blend with PRF and showed a sustained release of PDGF that stimulated cell activities efficiently.

In contrast, foam must not be used with PRF because it retains the supernatant hindering the presentation of growth factors and cytokines to the cells; in doing so, it hampers cell activities. Therefore, the use of foams as secondary dressing can abrogate PRF actions. Opposite to this, films and meshes do not retain the cytokine rich supernatant—the combination products shrink overtime and the supernatant can support cell activities. We observed reduced cell adhesion in the releasate from [Ag^+^-coated film + PRP] (data not shown); the presence of Ag^+^ in the releasate could potentially account for this reduction, which needs further confirmation [23].

In the clinical setting, the secondary dressing is often overlooked, probably because of the absence of significant differences with pairwise dressings’ comparisons [24]. In fact, whether the so-called active dressings are better than the conventional gauze remains to be clarified. In a clinical comparison between saline-moistened gauze and foam performed in 75 patients [25] the authors could not evidence any difference, thereby confirming previous studies [26]. Similarly, in a study with 50 patients, the gauze did not show any difference with foam [27]. Notwithstanding, according to the present study, differences between gauzes and foams are expected in the context of topical PRF therapies.

There is a broad range of dressing options and concepts in wound pathophysiology, which help to determine the type of dressing needed as well as the transition to a different dressing category. For example, diabetic foot ulcers are drier than venous ulcers, which in general are exudative. To assess the efficacy of various dressings in the management of diabetic ulcers, Saco M et al. [26] pooled data from 12 randomized controlled trials (RCTs) and performed a fixed effect metaanalysis with pairwise dressing comparisons. Merely three studies, including a total of 50 patients in the experimental group (hydrogel), have shown that hydrogel dressing was better than the conventional gauze. Nineteen RCTs examining dressing efficacies were identified in venous leg ulcers, comparing alginates, foams, hydrocolloids and gauzes. Only two studies found differences, and both compared hydrocolloid with paraffin gauze, but the risk of bias was high [28,29]. In the context of PRF, hydrocolloids differ from gauzes in the affinity index and in the ability to manage the releasate (they clustered differently), but both showed sustained release of the PDGF (representing the platelet cargo).

Because we are using PRP therapies in difficult-to-heal wounds, we undertook this study from a translational point of view, aiming to deliver useful information about the best dressing choice. In addition to the topical application of PRF, we also injected the ulcer edges with liquid PRP following the TIME acronym (Tissue, Infection, Moisture, Edges) for ulcer management, which emphasizes the importance of ulcer edges and surrounding tissues [30]. Moreover, PRF was re-applied in weekly intervals to cope with the instability of chemokines, cytokines and growth factors that provide the healing milieu.

With the present data in hand, the combination of PRF with polyurethane film is sound, as it allows gas exchange while not interfering with the native properties of PRF. Alternatively, depending on the ulcer bed, a dressing that blends PRF in efficient manner, i.e., alginate, hydrofiber or hydrocolloid, can help in the management of more exudative wounds. Nevertheless, additional measures to prevent damage in oversensitive skin in the perilesional area should be tailored for each patient. Therefore, the criteria of the clinical practitioners are still fundamental, although it is expected that the exposed in vitro results could help in the decision of choosing one dressing over the others, based on the specific characteristics of each patient.

In conclusion, wound dressings are intended to protect the ulcer bed without adhesion to the surface or decomposition for up to seven days. In this context, PRF can add biological activity to specific dressing types. The properties of the combination products [PRF + dressing] created thereby, particularly releasate management, affect GF availability and cell activities.

Adequate releasate handling is an important asset of hydrofibers and alginates, because they present moderate retention and slow release over seven days, which associate with PDGF-BB concentration and cell proliferation. In contrast, the usage of foams in the context of PRP therapies is not recommended because of their persistent retention of releasate; so, they hamper the interaction of signaling proteins with local and infiltrative cells and subsequent repair mechanisms.

On the other hand, combination products without any retention ability, such as [PRF + mesh], can favor lateral supernatant-flow wicking from the PRF, thus losing their efficacy.

Technologies have evolved from PDWHF to spraying technologies to deliver PRF or the use of combination products (i.e., PRP and Hyaluronan, Regen Lab SA, Mont-sur-Lausanne, Switzerland) [31]. At present, advanced therapies based on bioprinting technologies to create dermal constructs are being investigated. The preparation of bioinks is based on current knowledge about biomaterials, cells and signaling proteins active in wound healing. In this context, our data can orientate bioink engineering by taking advantage of the combination of biomaterials with patient-specific active healing cytokines from blood derivatives.

Our study has several limitations. We only examined the so-called active dressings as the advanced dressings with cellular products (e.g., DermagraftApligraf^®^, etc., Organogenesis, Canton, MA, US) were not under our pragmatic scope as they are too expensive to be used in a tertiary public hospital. Moreover, although there are a great number of collagen dressings which are moderately expensive, at present they are not available in our public health service. Furthermore, because of the complexity of healing mechanisms, which involve the interplay of immune, infiltrative and local cells, we only intended to obtain an estimate of dermal cell activities, enhancement of the whole healing process to be confirmed in animal models. To our knowledge, no RCT has explored this issue; however, the present in vitro data can help in the design of future trials paramount to make clinical recommendations.

## 4. Materials and Methods

**Figure 5:**
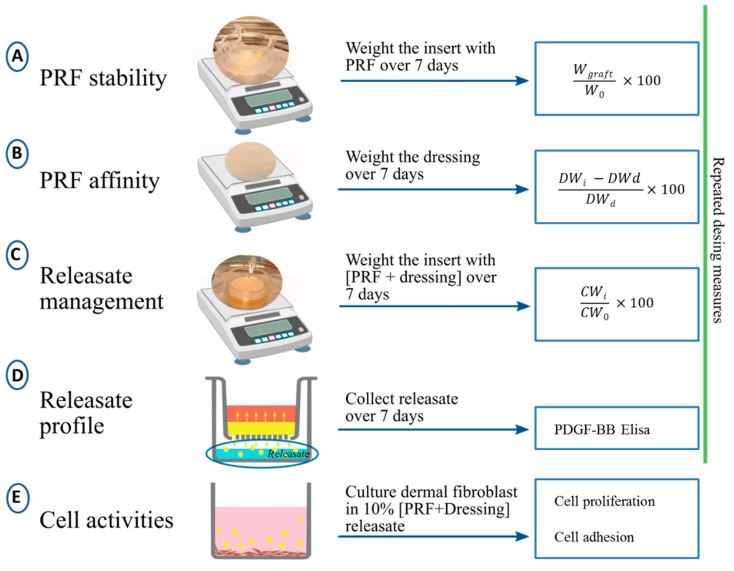
Experimental design. (**A**)Assessment of PRF stability; Wgraft: [insert + PRF] weight at each time point; W_0_: initial [insert + PRF] weight. (**B**) Assessment of PRF affinity index; DWi: dressing weight at different time point; DWd: dressing dry weight. (**C**) Releasate management measurement; CWi: [insert + combination product] weight at each time point; CW_0_: initial [insert + combination product] weight. (**D**) Kinetics of PDGF-BB release over 7 days; orange area: dressing; yellow area: PRF; yellow dots: releasate; yellow arrows: PRF releasate distribution; blue area: simulated wound fluid. (**E**) In vitro study of PRF realasate effect in cell activities; pink: cell culture conditioned media [DMEM + 10% PRF releasate]; yellow dots: releasate; bottom: adhered dermal fibroblasts.

The experimental set up is shown in Figure 5.

### 4.1. Platelet-Rich Fibrin (PRF)

The PRP was prepared from peripheral whole blood using sodium citrate as anticoagulant (Vacuette; Greiner BioOne, Kremsmünster, Austria). The donors of PRP in this study (six men and six women, age: 50.77 ± 6.35, range 37–60) were volunteers participating in a randomized control trial that responded positively to an informed consent of donation that was approved by the local ethics committee (CEIC 13/04). Briefly, the blood was centrifuged at 570 G for 7 min and the plasma layer was aspirated using a 5 mL syringe attached to 18 G-needle, under laminar flow following our standard operating procedures.

This PRP was the same that we used in our clinical studies and was pure PRP with moderate platelet enrichment (2.03 ± 0.78 times greater than peripheral baseline) and no leukocytes. According to the guidance from the Platelet Physiology Subcommittee of the Scientific Standardization Committee, the product was classified as PRP IIA1 [11].

PRF was formed by adding 10% CaCl_2_ to a final concentration 22.5 mM, incubating 15 min at 37 °C in a glass crystallizer, [2 mL PRP + 100 µL CaCl_2_].

The active molecules in PRP releasate were assessed using solid-phase sandwich enzyme- linked immunoabsorbent assays (ELISAs) and solid-phase enzyme-amplified sensitivity immunoassays (EASIA). The ELISA procedures were used to quantify hepatocyte growth factor (R&D Systems Inc, Minneapolis, MN, USA), VEGF (−165) (PeproTech House (London, UK), GRO-alpha/CXCL1, and MCP-1/CCL2 (Ray-Biotech Inc, Norcross, GA, USA). The EASIA procedures were used to quantify RANTES/CCL5 (Biosource Europe SA, Nivelles, Belgium). All kits were used according to the manufacturer’s instructions.

### 4.2. Stability of PR-Fibrin Graft

To examine the PRF stability, analyses were done for each donor separately in duplicate. Inserts containing the PRFs were weighted over seven days. Changes in PRF, due to releasate extrusion, were calculated using these measures (minus insert weight) related to initial weight W_0_, (Wgraft/Wo) × 100 = remaining weight (%) over time.

### 4.3. Interaction of Different Wound Dressing Classes with PRF

Grounded in the clinical practice in our hospital, we selected representative dressings for each of the following seven categories: transparent polyurethane film (Tegaderm™ Film, 3M Health Care), foam (Mepillex^®^ Border, Mölnlycke Health Care, Gothenburg, Sweden), alginate (Melgisorb^®^Plus, Mölnlycke Health Care, Gothenburg, Sweden), hydrofiber (Aquacel™ extra™, ConvaTec Ltd., Reading, Berkshire, UK), hydrocolloid (Varihesive^®^ Gel Control, ConvaTec Ltd., Reading, Berkshire, UK), mesh (Acticoat^®^ Flex 3, Smith and Nephew, Watford, UK) and non-impregnated polymer gauze (Texpla^®^ Texpol, Manresa, Barcelona, Spain). Table S1 (Supplementary data) shows the dressing products, composition and main properties.

For the study of combination products, i.e., [PRF graft + wound dressings], 3.8 cm^2^ diameter sections of each dressing type were cut using a 3D-printed template.

To obtain a representative PRF graft, PRPs from 12 donors were pooled and calcified. Next, 2 mL aliquots were placed in glass crystallizers and incubated until PRF formation. Thereafter, PRFs were placed on transwell inserts and various types of dressings deposited on the top. All samples were kept at 37 °C, 5% CO_2_ until examination. The experiments were performed in duplicate for each of the combination products, [PRF + wound dressings].

#### 4.3.1. Affinity of Different Wound Dressing Types for PRF

Dressings from the different categories were tested for PRF affinity over seven days (at 0, 1, 2, 3, 4, 5 and 7 days) following the protocol previously described by Faramazi et al. [32]. The ability of the various dressings to keep PRF (affinity index) was calculated using the following formula: [(DWi − DW_d_)/DW_d_], DW_d_ = Dressing dry weight; DW_i_ = dressing weight at different time points.

#### 4.3.2. Assessment of Releasate Management (Retention/Extrusion)

The inserts containing the combination products were weighted at t = 0 and over 7 days (at 1, 2, 3, 4, 5 and 7 days). Releasate handling (% mass retained/lost) was calculated using these measures related to initial weight, (CWi/CWo) × 100, ( CWi: [insert + combination product] weight at each time point; CW_0_: initial [insert + combination product] weight).

### 4.4. Assessment of the Release Profile of PDGF-BB

In order to assess the kinetics of PDGF-BB release for each [PRF + dressing], the combination products were placed on the upper chamber and 300 µL of simulated wound fluid (WF: [142 mM] NaCl and [2.5 mM] CaCl_2_ [33]) were added to each bottom well.

During the incubation period, the total volume of extruded supernatant was collected at 1, 3 and, 24 h and 2, 3, 4 and 7 days for each condition and stored at −20 °C before analysis. At every time-point, 300 µL of fresh WF were added back into every well.

The concentration of PDGF-BB was assessed by the Human PDGF-BB Standard TMB ELISA Development kit (Peprotech Inc, Rocky Hill, NJ, USA).

### 4.5. Cell Culture

Human primary adult dermal fibroblasts were purchased from Lonza (Basel, Switzerland) and were cultured in DMEM/F12 (ThermoFisher Scientific, Waltham, MA, USA) at 37 °C and 5% CO_2_ in a humidified atmosphere. Cells were grown to ~80% confluence and harvested using a trypsin–EDTA solution. For these experiments, cells were used between the passages 4–8.

### 4.6. The Influence of Combination Products’ Releasates in Cell Behavior

#### 4.6.1. Cell Proliferation Assay

Fibroblast cultures at a confluence of ~80% were starved in serum-free media for 24 h. A suspension of 250 cell/mL of 24 h starved cells diluted in each [PRF + dressing] conditioned media (DMEM/F12 + 10% of the supernatant released after 24 h from each combination product [PRF + dressing]) were placed in 96-well plates in quadruplicates. After culturing the cells for 24, 48 and 72 h, XTT (2,3-bis(2-methoxy-4-nitro-5-sulfophenyl)-5-[(phenylamino) carbonyl]-2H-tetrazolium hydroxide) solution (Sigma-Aldrich, St. Louis, MO, USA) was added to each well in order to assess the influence of the releasate of each dressing in the proliferation of fibroblast. Following 4 h incubation with XTT, the absorbance of each well was determined in a spectrophotometer at 490 nm.

#### 4.6.2. Cell Adhesion Assay

The fibroblasts were seeded in 96-well plates following the same protocol as for cell proliferation. One hour after deposition, the culture media of each well was aspirated to eliminate non-adhered cells, and the presence of adhered cells was studied using XTT.

### 4.7. Statistical Analyses

The data are shown as mean ± SD. To examine the differences and similarities of various dressing types with PRF, we performed hierarchical cluster analyses using Ward’s method and the squared Euclidian distance between cluster centers. Hierarchical relationships between dressings are represented as dendograms. The clustered groups were compared using repeated measures ANOVA and eta squared as a measure of the effect size. Linear regressions were used to examine the influence of dressing properties on cell behavior. A cubic transformation of the 24 h releasate was performed to linearize its relationship with proliferation.

## Figures and Tables

**Table 1:** PRP composition in terms of cell counts.

Cell Count		Whole Blood	Platelet-Rich Plasma
Leukocytes	(× 10^−3^/µL)	5.2 ± 0.87	0.06 ± 0.07 (lymphocytes)
Erythrocytes	(× 10^−6^/µL)	4.17 ± 0.48	n.d.
Platelets	(× 10^−3^/µL)	220 ± 0.42	460 ± 103

n.d: non detected.

**Table 2:** The concentration of relevant molecules involved in healing mechanisms in platelet-rich fibrin PRF releasates.

Active Molecules	Concentration in PRF Releasate	Range
MCP-1	524 pg/mL	100–1440
VEGF	166 pg/mL	120–260
HGF	612 pg/mL	430–870
RANTES	24 ng/mL	22–27
GRO-α	900 pg/mL	80–2370
PDGF-BB	12 ng/mL	2–18

MCP: monocyte chemoattractant protein; VEGF, vascular endothelial growth factor; HGF, hepatocyte growth factor; RANTES, regulated upon activation, normally T-expressed and presumably secreted; GRO-α, neutrophil activating protein 3; PDGF-BB, platelet-derived growth factor.

## Supplementary Materials

Supplementary materials can be found at https://www.mdpi.com/1422-0067/21/2/624/s1.
