# Supplementary material for: Wound Dressing Selection Is Critical to Enhance Platelet-Rich Fibrin Activities in Wound Care
Source: Int J Mol Sci. 2020 Jan 17;21(2):624. doi: 10.3390/ijms21020624 (PMC7013388; doi:10.3390/ijms21020624)
Supplement: Supplementary file 1 [file ijms-21-00624-s001.pdf]

SUPPLEMENTARY MATERIALS

Table 1. Dressing Products, composition and main properties.

Type of dressing	Composition	Main properties	Commercially available, (Manufacturer)
**FILM**	Transparent film dressing, polyurethane film	Adherent, permeable to water vapor, oxygen and carbon dioxide, impermeable to bacteria and water	Tegaderm ™ Film (3M Helath Care)
**FOAM**	Trilaminar structure containing polyurethane, PEG, silicone	Moderately absorbent, fluid retention, semi-occlusive, insulating, permeable to water and gas	Mepillex ® Border (Mölnlycke Health Care)
**HYDROCOLLOID**	Hydrocolloid (CMC, pectin, gelatin) Polyurethane waterproof outer layer	Absorbs exudates and swells into a hydrophilic gel, fluid trapping, keeps wound moist, occlusive, long-time between changes, promotes granulation tissue	Varihesive® Gel Control (ConvaTec Ltd)
**HYDROFIBER**	CMC fibers	Highly absorbent, forms a gel inside wound bed, keeps wound moist, traps microbes inside the gel	Aquacel™ extra ™ (ConvaTec Ltd)
**ALGINATE**	Alginic acid, nonwoven fibers covered with Ca^{2+}	Highly absorbent, forms a hydrophilic gel inside wound bed, keeps wound moist, hemostatic through Ca^{+2} release	Melgisorb® plus (Mölnlycke Health Care)
**MESH**	Flexible polyester with Ag^{+} nanocristals	Low adherence, bactericide, sustained Ag^{+} release	Acticoat◊ Flex 3 (Smith&Nephew)
**GAUZE**	Gauze - 100% nonwoven synthetic fiber (polyester)	Hydrophilic, absorbent, inexpensive	Texpla® (Texpol)
